# Distinct Bacterial Pathways Influence the Efficacy of Antibiotics against Mycobacterium tuberculosis

**DOI:** 10.1128/mSystems.00396-20

**Published:** 2020-08-04

**Authors:** Michelle M. Bellerose, Megan K. Proulx, Clare M. Smith, Richard E. Baker, Thomas R. Ioerger, Christopher M. Sassetti

**Affiliations:** a Department of Microbiology and Physiological Systems, University of Massachusetts Medical School, Worcester, Massachusetts, USA; b Department of Computer Science and Engineering, Texas A&M University, College Station, Texas, USA; Marquette University

**Keywords:** antibiotic treatment, host-microbe interactions, microbial genomics, *Mycobacterium tuberculosis*

## Abstract

Effective tuberculosis treatment requires at least 6 months of combination therapy. Alterations in the physiological state of the bacterium during infection are thought to reduce drug efficacy and prolong the necessary treatment period, but the nature of these adaptations remain incompletely defined. To identify specific bacterial functions that limit drug effects during infection, we employed a comprehensive genetic screening approach to identify mutants with altered susceptibility to the first-line antibiotics in the mouse model. We identified many mutations that increase the rate of bacterial clearance, suggesting new strategies for accelerating therapy. In addition, the drug-specific effects of these mutations suggested that different antibiotics are limited by distinct factors. Rifampin efficacy is inferred to be limited by cellular permeability, whereas isoniazid is preferentially affected by replication rate. Many mutations that altered bacterial clearance in the mouse model did not have an obvious effect on drug susceptibility using *in vitro* assays, indicating that these chemical-genetic interactions tend to be specific to the *in vivo* environment. This observation suggested that a wide variety of natural genetic variants could influence drug efficacy *in vivo* without altering behavior in standard drug-susceptibility tests. Indeed, mutations in a number of the genes identified in our study are enriched in drug-resistant clinical isolates, identifying genetic variants that may influence treatment outcome. Together, these observations suggest new avenues for improving therapy, as well as the mechanisms of genetic adaptations that limit it.

**IMPORTANCE** Understanding how Mycobacterium tuberculosis survives during antibiotic treatment is necessary to rationally devise more effective tuberculosis (TB) chemotherapy regimens. Using genome-wide mutant fitness profiling and the mouse model of TB, we identified genes that alter antibiotic efficacy specifically in the infection environment and associated several of these genes with natural genetic variants found in drug-resistant clinical isolates. These data suggest strategies for synergistic therapies that accelerate bacterial clearance, and they identify mechanisms of adaptation to drug exposure that could influence treatment outcome.

## Abstract (precis)

Understanding how Mycobacterium tuberculosis survives during antibiotic treatment is necessary to rationally devise more effective tuberculosis (TB) chemotherapy regimens. Using genome-wide mutant fitness profiling and the mouse model of TB, we identified genes that alter antibiotic efficacy specifically in the infection environment and associated several of these genes with natural genetic variants found in drug-resistant clinical isolates. These data suggest strategies for synergistic therapies that accelerate bacterial clearance, and they identify mechanisms of adaptation to drug exposure that could influence treatment outcome.

## INTRODUCTION

The current regimen for tuberculosis (TB) chemotherapy was developed through a series of large clinical trials in the early 1970s (1). The resulting “short-course regimen” consists of four drugs, isoniazid (INH), rifampin (RIF), pyrazinamide (PZA), and ethambutol (EMB) (2). Combining these agents reduced the duration of treatment from 12 to 18 months to as little as 6 months (3). The wide-scale application of this regimen is generally considered a public health success and is estimated to have cured over 50 million patients in the last 2 decades (4). Despite this success, delivering the extended therapy necessary to prevent recurrent disease is difficult in many settings and TB remains a leading cause of infectious death worldwide (4). The rational design of more rapid and effective therapies would be facilitated by understanding the mechanisms that limit the efficacy of our current drugs.

It has been clear since the first animal treatment studies that the requirement for prolonged therapy correlates with the relatively slow killing of Mycobacterium tuberculosis in the host (5, 6). Both INH and RIF are rapidly bactericidal in laboratory culture, but these agents clear bacteria much more slowly from the lungs of infected animals (7). While drug penetration into TB lesions can be limiting (8), suboptimal drug exposure alone is unlikely to fully account for persistence of viable bacteria. In addition, bacterial adaptations to the host environment have been proposed to limit drug efficacy via a number of mechanisms. For example, the rate at which most antibiotics kill is related to growth rate and metabolic activity of bacteria (9–11), and the relatively slow replication of M. tuberculosis during infection correlates with reduced drug efficacy (12). More specific adaptations to this environment, such as the induction of stress responses (13), changes in cell wall permeability (14), and expression of efflux pumps (15), have also been proposed to play an important role.

In addition to these inducible adaptations to the host environment, the widespread application of TB chemotherapy has also selected for stable genetic variants that promote bacterial survival. Most obviously, strains harboring high-level resistance conferring mutations in drug targets or prodrug activators have become common (16). The resulting “resistance” phenotype increases the MIC of the corresponding antibiotic. Recent studies have shown that even small changes in MIC can negatively affect treatment outcome (17). In addition, recent bacterial genome-wide association studies (GWAS) have identified genetic variants that are associated with drug resistance but do not directly affect MIC. Some of these mutations compensate for the fitness cost imposed by primary resistance-conferring variants (18). In other cases, mutations may promote prolonged bacterial survival in the presence of antibiotic (19), a phenotype termed drug “tolerance.” While hundreds of drug resistance-associated variants have been described (19–22), the vast majority have not been functionally characterized.

In order to more globally define bacterial pathways that alter drug efficacy during infection, we designed a study to identify efficacy-altering mutations directly during infection using transposon sequencing (TNseq) in an animal model of TB. TNseq provides an unbiased approach to study conditional gene essentiality by comprehensively comparing the effects of loss-of-function mutations in different environments. Unlike previous studies that focused on individual mechanisms that broadly alter drug efficacy *in vitro* (23–25), our unbiased study found that most efficacy-altering mutations are antibiotic specific, are unrelated to growth rate, and alter the effect of antibiotics only in the *in vivo* environment. A number of these efficacy-altering genes harbor mutations that are associated with drug resistance in clinical M. tuberculosis isolates, indicating that similar mechanisms may influence treatment outcome.

## RESULTS

### Selection of transposon mutant libraries in antibiotic-treated mice.

**FIG 1:**
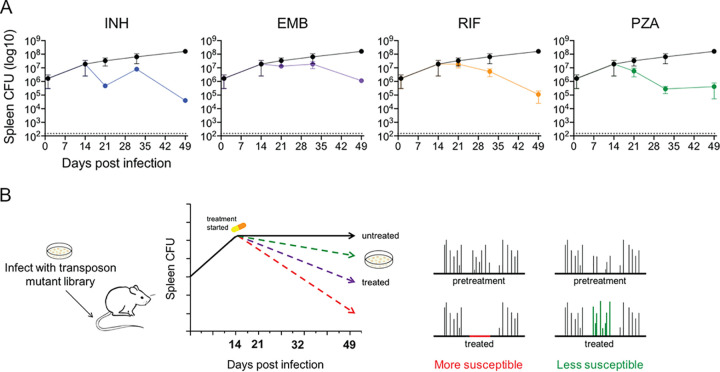
Genetic strategy to identify mutations that alter susceptibility to antibiotic treatment in mice. (A) Spleen CFU from BALB/c mice infected with transposon mutant library either untreated (black circles) or treated with indicated antibiotic. Treatment was started at 14 days postinfection. Mean and standard deviation from biological replicates are plotted (*n* = 2 to 7 per time point). (B) Diagram of TNseq screen design. BALB/c mice were infected via intravenous route with 2 × 10^6^ bacteria/mouse. At 14 days postinfection, pretreatment libraries were collected, via plating, and treatment regimens were initiated. Time points were collected, via plating, from untreated and treated mice at 21, 32, and 49 days postinfection. Comparison of transposon insertion abundances pre- and posttreatment identifies mutants more susceptible (decrease in insertions) and less susceptible (increase in insertions).

A differential selection strategy was designed to identify bacterial mutants that alter the efficacy of each of the first-line TB therapeutics, INH, EMB, RIF, and PZA. Mice were infected with a complex transposon mutant library representing >50,000 independent insertion events via the intravenous (i.v.) route. The infection was allowed to progress for 2 weeks to establish the adaptive immune responses that accentuate drug tolerance (26). We initially assessed bacterial survival in the spleen, since the representation of the entire library could be maintained in each individual mouse at this site. Spleen infection is a model of intracellular growth in the presence of adaptive immunity, a combination of conditions that resembles many aspects of the primary pulmonary site of infection (27–29). At the initiation of drug treatment, the bacterial population had expanded to an average of 2 × 10^7^ CFU/spleen. As expected, different antibiotics cleared the bacteria at distinct rates (Fig. 1A). However, each drug, even the bacteriostatic agent, EMB, significantly reduced bacterial burden over 5 weeks of therapy. At this time point, all drugs had reduced the bacterial burden by >100-fold, but relatively diverse libraries could still be recovered. Similar rates of clearance were observed in the lung (see Fig. S1 in the supplemental material). Only in PZA-treated mice did we observe a decreased rate of killing between 2.5 and 5 weeks, suggesting the possible expansion of resistant clones.

10.1128/mSystems.00396-20.1 FIG S1 Lung CFU from transposon mutant library infection. Lung CFU from BALB/c mice infected with transposon mutant library via intravenous route, 2 × 10^6^ bacteria/mouse, either untreated (black circles) or treated with indicated antibiotic. Treatment started at 14 days postinfection. Plotted mean and standard deviation from biological replicates (*n* = 2 to 7 per time point). Download FIG S1, TIF file, 0.3 MB. Copyright © 2020 Bellerose et al. 2020 Bellerose et al. This content is distributed under the terms of the Creative Commons Attribution 4.0 International license.

To identify genes that alter bacterial fitness in this environment, we used TNseq to quantify the relative abundance of each transposon mutant in libraries recovered before infection; immediately before the initiation of therapy; from mice treated for 1, 2.5, or 5 weeks; or from untreated mice at the same time points (Fig. 1B). Surviving bacteria were recovered from the spleen of each mouse by plating. Transposon-chromosome junctions in chromosomal DNA were ligated to unique molecular identifiers (UMI), amplified, and sequenced (30). The relative abundance of each mutant in a pool was estimated based on the number of corresponding UMI sequences. This design allowed the independent quantification of mutant fitness under the pressures imposed by the host and by the combined pressure of host immunity and antibiotic therapy.

### Identification of genes necessary for bacterial fitness in untreated animals.

**FIG 2:**
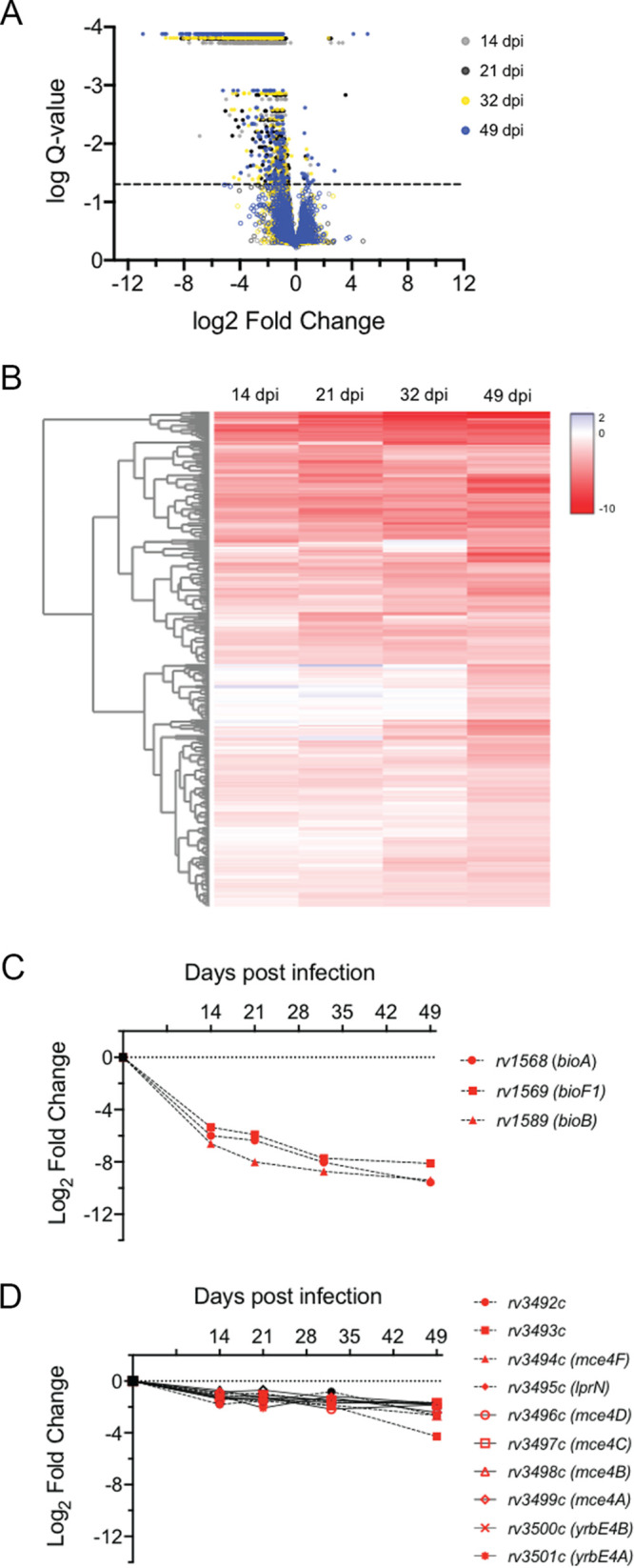
Genes required for optimal fitness *in vivo*. (A) Volcano plot of *in vivo* libraries compared to *in vitro* input library at indicated time points (dpi, day postinfection). Q-value <0.05 is indicated by the dashed line. Genes meeting significance (Q-value <0.05) are indicated by filled circles. (B) Heatmap of the relative abundance of 562 genes significantly underrepresented *in vivo* at each time point. Genes are hierarchically clustered based on log_2_ fold change at individual time points. (C and D) TNseq phenotype of genes/operons significantly underrepresented *in vivo* at each time point: biotin biosynthesis genes (C) and *mce4* operon (D). Significance (Q-value <0.05) is indicated by red symbols.

Initially, TNseq libraries recovered from the untreated mice were analyzed to determine the relative fitness of each mutant over the time course of our infection. Libraries recovered at each time point were compared to the input libraries used for the infection (Table S1). In total, 562 genes were found to be required for optimal fitness *in vivo* by 49 days postinfection (Fig. 2A and Table S2). We observe up to 77% overlap with genes previously reported to be required for replication in the mouse model using similar approaches (Table S2) (27, 29, 31). These genes encode a wide variety of functions previously verified to be necessary for replication in mice, including type VII protein secretion (ESX1), cholesterol (Mce4) and fatty acid (Mce1) catabolism, and siderophore transport (IrtAB, MmpL4/S4). The 231 novel genes identified in our study likely reflect the longer period of infection and more accurate quantification that resulted from the greater number of animals used.

10.1128/mSystems.00396-20.5 TABLE S1 Whole-genome phenotypic profiling of M. tuberculosis mutants *in vivo.* Pairwise analyses of *in vivo* libraries compared to *in vitro* input libraries. “Mean TAs hit,” average number of transposon insertion sites sequenced across all comparisons. “Mean_hits/TA,” average number of unique sequenced templates divided by the “TA” insertion sites in the gene. “Pval,” *P* value derived from resampling. “Qval,” significance adjusting for multiple tests. Gray shading, low-confidence genes (having an average of 3 or fewer transposon insertions across all comparisons). Download Table S1, XLSX file, 1.2 MB. Copyright © 2020 Bellerose et al. 2020 Bellerose et al. This content is distributed under the terms of the Creative Commons Attribution 4.0 International license.

10.1128/mSystems.00396-20.6 TABLE S2 Significantly underrepresented genes *in vivo.* Comprehensive list of genes significantly underrepresented (Q-value <0.05) postinfection compared to *in vitro* input library at any time point. Genes with <4 TAs and <4 TAs with insertions were excluded from this list. “Qval,” significance adjusting for multiple tests. Red = log_2_ ≥−1.5. Bold = Qval <0.05. Download Table S2, XLSX file, 0.08 MB. Copyright © 2020 Bellerose et al. 2020 Bellerose et al. This content is distributed under the terms of the Creative Commons Attribution 4.0 International license.

The availability of time course data allowed the assessment of mutant fitness at different stages of infection. The 2-week time point captures the early expansion of the bacteria, before the onset of the adaptive response. The later time points reflect additional pressures imposed by T cells that control bacterial replication. As expected, we observed a progressive depletion of mutants over this time course (Fig. 2B), and distinct sets of genes were found to be important in establishing infection or persisting at later time points. For example, biotin biosynthetic mutants were dramatically underrepresented at the earliest time points, reflecting their known inability to replicate *in vivo* (32) (Fig. 2C). In contrast, Mce4 mutants were well represented at the early time point but became progressively depleted from the pool, reflecting their specific deficit in fitness upon onset of adaptive immunity (33) (Fig. 2D). These data validated our methodology and provide insight into the stresses that M. tuberculosis may encounter during different times of infection independent of drug treatment.

### Identification of mutants with altered susceptibility to antibiotics.

**FIG 3:**
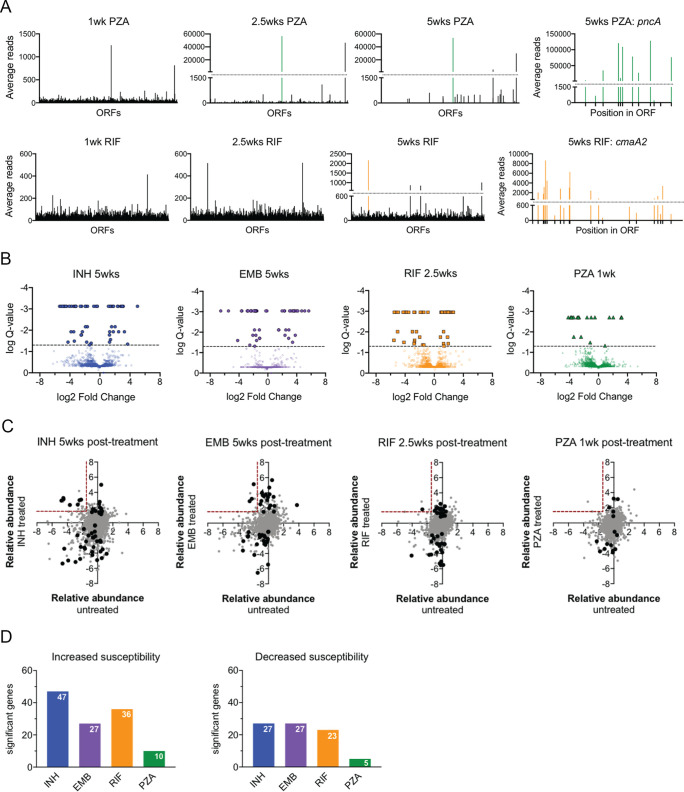
Mutants with altered susceptibility to antibiotics. (A) Average reads of transposon insertions for each open reading frame (ORF) in the H37Rv genome. (Top) PZA-treated libraries; *pncA* is indicated by the green line; average reads for each TA dinucleotide site in *pncA* at 5 weeks posttreatment. (Bottom) RIF-treated libraries; *cmaA2* is indicated by orange line; average reads for each TA dinucleotide site in *cmaA2* at 5 weeks posttreatment. (B) Volcano plots of treated libraries at individual time points compared to pretreatment libraries. Treatment lengths indicated by symbol: triangles, 1 week; squares, 2.5 weeks; circles, 5 weeks. Negative log_2_ fold change = underrepresented posttreatment. Positive log_2_ fold change = overrepresented posttreatment. Q-value <0.05 is indicated by the dashed line. Genes meeting significance (Q-value <0.05) are indicated by filled symbols. (C) The relative abundance, log_2_ fold change, for each gene in untreated libraries (*x* axis) or treated libraries (*y* axis). Genes significantly altered posttreatment are in black. The red dashed line indicates the threshold for genes that are attenuated *in vivo* and less susceptible to antibiotic treatment. (D) The number of genes with a significant decrease in transposon insertions (left) and a significant increase in transposon insertions (right), under each treatment condition.

A critical requirement for TNseq-based comparisons is maintaining the complexity of each library to reduce stochastic effects. Treatment decreases the number of viable bacteria, which could result in decreased representation of mutants across the genome. As a result, we first assessed the complexity of the libraries recovered from drug-treated mice. Initial analyses, calculating the average reads derived from transposon insertions in each gene, indicated that libraries exposed to extended RIF or PZA treatments were less complex than the rest (Fig. 3A and Fig. S2). This effect was particularly clear for PZA, where the library became dominated by mutants with a disrupted *pncA* gene, which encodes the activator for the PZA prodrug. A similar, but less pronounced, effect was found upon RIF treatment, where mutants with mutations in the *cmaA2* gene became the most abundant strains in each sample from extended RIF treatments. The *cmaA2* gene encodes a cyclopropane synthase which modifies the mycolate layer of the cell wall and alters cellular permeability (34). In both cases, transposon insertions throughout these genes were enriched, indicating that the loss of gene function was responsible for altering drug efficacy. While a small number of other mutants appeared to be enriched upon extended therapy, these were not consistent between samples and represented single insertion events, likely reflecting the presence of spontaneous resistance-conferring mutations that are unlinked to the insertion. Thus, the lack of complexity in these libraries led to the exclusion of long-term RIF and PZA samples from the following comparative analyses.

10.1128/mSystems.00396-20.2 FIG S2 Assessment of mutant abundance in antibiotic treated libraries. (A) Genome view of transposon insertions. Average reads of transposon insertions for each ORF in the H37Rv genome. (Top) INH. (Bottom) EMB. (B) Comparison of untreated and posttreatment mutant phenotypes. Relative abundance, log_2_ fold change, for each gene in untreated libraries (*x* axis) or treated libraries (*y* axis). Genes significantly altered posttreatment are in black. The red dotted line indicates the threshold for genes that are attenuated *in vivo* and less susceptible to antibiotic treatment. Download FIG S2, TIF file, 0.7 MB. Copyright © 2020 Bellerose et al. 2020 Bellerose et al. This content is distributed under the terms of the Creative Commons Attribution 4.0 International license.

We next compared mutant abundance between pre- and posttreatment samples to quantify mutant survival during therapy (Fig. 3B and Table S3). We first compared mutant fitness in treated versus untreated animals, by comparing each time point to the pretreatment sample, to estimate the relationship between replication rate and drug efficacy (Fig. 3C). We observed the most overlap in the context of INH, a drug with clear growth-rate-dependent effects *in vitro* (35). However, this effect was not apparent for other drugs (Fig. 3C and Fig. S2), indicating that the drug tolerance phenotypes we observed were not primarily related to changes in growth rates of mutants.

10.1128/mSystems.00396-20.7 TABLE S3 Whole-genome phenotypic profiling of antibiotic-treated M. tuberculosis mutants *in vivo*. Pairwise analyses of *in vivo* antibiotic-treated libraries compared to the *in vivo* pretreatment libraries. “Mean TAs hit,” average number of transposon insertion sites sequenced across all comparisons. “Mean_hits/TA,” average number of unique sequenced templates divided by the “TA” insertion sites in the gene. “Pval,” *P* value derived from resampling. “Qval,” significance adjusting for multiple tests. Gray shading, low-confidence genes (having an average of 3 or fewer transposon insertions across all comparisons). Download Table S3, XLSX file, 3.0 MB. Copyright © 2020 Bellerose et al. 2020 Bellerose et al. This content is distributed under the terms of the Creative Commons Attribution 4.0 International license.

We next used a nonparametric resampling strategy to identify mutants that were differentially represented in pre- and posttreatment pools. For each antibiotic regimen, we observed mutants that were both under- and overrepresented in the posttreatment samples (Fig. 3D). The genes identified are involved in a range of functions and include pathways known to alter antibiotic efficacy. For example, *pncA* and *glpK* mutants were found to be less sensitive to PZA treatment, consistent with previous studies (36). Conversely, we identified mutants in *ppe50/51*, which had previously been shown to increase the efficacy of a multidrug regimen (36). We also identified multiple mutants lacking putative antibiotic efflux pumps, including ABC transporters Rv1747 and Rv1273, which were more susceptible to INH and RIF, respectively. Overall, we found 160 mutants that altered efficacy of antibiotic treatment (Table S4).

10.1128/mSystems.00396-20.8 TABLE S4 Genes with significantly altered susceptibility *in vivo*. Comprehensive list of genes with significantly altered susceptibility to antibiotic treatment (Q-value <0.05) *in vivo* compared to pretreatment control libraries under any condition. Genes with <4 TAs and <4 TAs with insertions were excluded from this list. “Qval,” significance adjusting for multiple tests. Green = log_2_ ≥1.5. Red = log_2_ ≥−1.5. Bold = Qval <0.05. Download Table S4, XLSX file, 0.05 MB. Copyright © 2020 Bellerose et al. 2020 Bellerose et al. This content is distributed under the terms of the Creative Commons Attribution 4.0 International license.

### Validation of mutant phenotypes in an aerosol infection model.

To determine how well the TNseq study predicted the phenotype of loss-of-function mutations, a series of deletion mutants were generated. These genes were selected based on statistical criteria that consider each distinct transposon insertion in a gene to be an independent assessment of the loss-of-function phenotype. As a result, genes that are predicted to alter drug efficacy contain a number of independent insertions that all produce a similar effect (Fig. S3). We also included mutants that disrupt different cellular functions and produce both qualitatively and quantitatively distinct phenotypes. For this analysis we included previous TNseq data from mice treated with the combination regimen HRZE (consisting of INH, RIF, PZA, and EMB) using a parallel treatment regimen (36).

10.1128/mSystems.00396-20.3 FIG S3 Transposon insertions pre- and posttreatment in genes with altered susceptibility. Average number of unique sequencing reads (*y* axis) plotted versus TA dinucleotide sites (*x* axis) for pretreatment (black) and posttreatment (INH, blue; EMB, purple; RIF, orange; PZA, green). Plotted mean and standard deviation from biological replicates. (A) *rv3822*; (B) *rv1184c*; (C) *rv1174c*; (D) *cinA*; (E) *rv1747*; (F) *rv1273c*; (G) *ppe51*; (H) *rv0248c*; (I) *cmaA2*. Download FIG S3, TIF file, 1.3 MB. Copyright © 2020 Bellerose et al. 2020 Bellerose et al. This content is distributed under the terms of the Creative Commons Attribution 4.0 International license.

**FIG 4:**
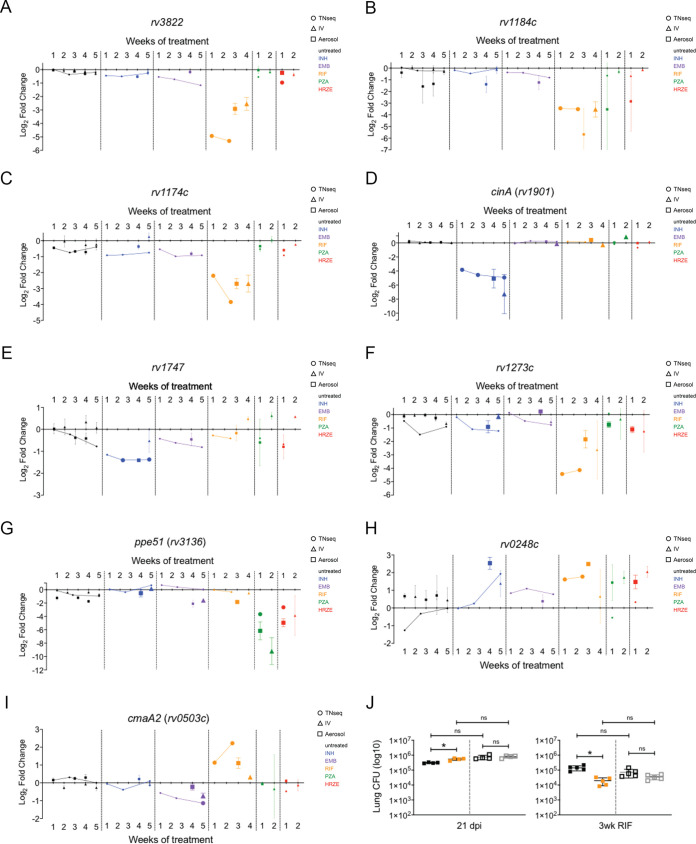
Validation of mutant phenotypes. (A to I) The relative abundance, log_2_ fold change, of mutants after TNseq (circles), i.v. (triangles), or aerosol (squares) infections. Mean and standard deviation from biological replicates for i.v. and aerosol infections are plotted. Significance is indicated by increased symbol size: TNseq, resampling Q-value <0.05; i.v. and aerosol, unpaired *t* test with Benjamini-Hochberg multiple testing correction, *P* < 0.05. Conditions are indicated by color: untreated (black), INH (blue), EMB (purple), RIF (orange), PZA (green), HRZE (red). HRZE data were obtained from reference 36. (J) Lung CFU of H37Rv (black), Δ*rv1273c* (orange), and complement (gray) strains after aerosol infection and treatment with RIF. Data represent two aerosol infections of combined bacteria, 1:1 Rv and Δ*rv1273c* mutant (filled squares) and 1:1 Rv and complement (open squares). Mean and standard deviation are plotted; individual points are biological replicates. Significance was determined using unpaired *t* test with Benjamini-Hochberg multiple testing correction: * (0.03), ** (0.002), and *** (0.0002). ns, not significant.

Individual deletion strains were constructed to contain a barcode at the site of deletion which served as an identifier for downstream quantification via sequencing. To measure susceptibility of the knockout strains, mutant and wild-type strains were mixed into a pool of nine strains for infection via either intravenous (i.v.) or aerosol routes. Treatment was initiated at 2 weeks postinfection, and the duration for individual antibiotics was adjusted to produce a similar decrease in CFU for each of the bactericidal regimens and to maintain library complexity (Fig. S4). At indicated time points, bacteria were isolated via plating the spleen or lung for i.v. and aerosol infections, respectively. The abundance of each mutant relative to wild type was calculated and normalized to their pretreatment abundance, allowing a direct comparison to the TNseq data (Fig. 4A to I).

10.1128/mSystems.00396-20.4 FIG S4 CFU of pooled intravenous and aerosol infections. Plotted mean and standard deviation; individual points are biological replicates. Treatment condition indicated by color: pretreatment (black), INH (blue), EMB (purple), RIF (orange), PZA (green), and HRZE (red). Dashed line indicates target killing for bactericidal antibiotics. (A) Spleen CFU from BALB/c mice after intravenous infection. Treatment started at 14 days postinfection. Data represent two infections indicated by closed and open triangles. (B) Lung CFU from BALB/c mice after aerosol infection. Treatment started at 21 days postinfection. Data represent two infections indicated by closed and open squares. Download FIG S4, TIF file, 0.3 MB. Copyright © 2020 Bellerose et al. 2020 Bellerose et al. This content is distributed under the terms of the Creative Commons Attribution 4.0 International license.

In nearly every case, the altered susceptibility phenotypes predicted by TNseq were reproduced using deletion mutants upon i.v. and/or aerosol infection. Many mutants were predicted to enhance the efficacy of individual antibiotics. These included genes that were among the 10 strongest hypersusceptible phenotypes for RIF (*rv1184c*, *rv3822*, and *rv1174c*) and INH (*cinA*) (Fig. 4A to D). Additionally, mutations affecting two ABC transporters, Rv1747 and Rv1273c, suggested that these proteins could function as efflux pumps for INH and RIF, respectively (Fig. 4E and F). PZA-specific effects were observed as well. We confirmed that *ppe51* mutant strains have increased susceptibility to PZA-containing regimens (Fig. 4G), consistent with previous work (36). Other mutations were predicted to decrease efficacy. For example, mutants lacking the succinate dehydrogenase component, Rv0248c, were consistently cleared less rapidly than wild-type bacteria. This phenotype was observed upon treatment with different regimens (INH, RIF, and HRZE), suggesting that this mutation produces tolerance to many unrelated antibiotics (Fig. 4H). CmaA2 mutants were predicted to have a complex phenotype, with opposing susceptibilities to EMB and RIF (Fig. 4I). We validated CmaA2 mutants as more susceptible to EMB treatment, consistent with previous studies (34), and less susceptible to RIF, as we observed in our initial analyses of library complexity (Fig. 3). These opposing phenotypes may compensate for each other during combination therapy, as we observe a neutral phenotype in the HRZE regimen.

To assess whether the relative abundance determined by sequencing mutant pools reflected genuine differences in viable bacteria, we mixed the Δ*rv1273c* putative efflux pump mutant strain and its complemented strain and performed additional infections using CFU as a measure of abundance. Using a competitive model in which each mutant was mixed at a 1:1 ratio with wild type and inoculated via the aerosol route, we observed that the Δ*rv1273c* mutant was cleared more rapidly from the lung than wild type or the complemented strain by RIF treatment (Fig. 4J), as anticipated. We conclude that the TNseq data provide an accurate assessment of relative mutant abundance in this system.

### Mutations produce drug-specific effects.

**FIG 5:**
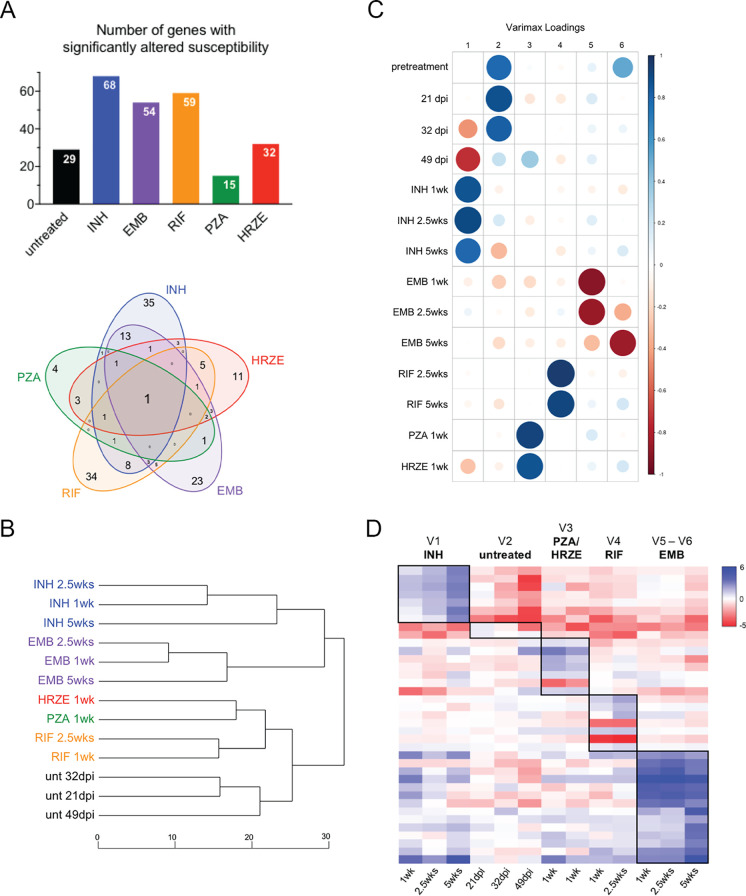
Mutations associated with individual antibiotic treatments. (A) (Top) The number of genes with a significant change in transposon insertions under each condition. (Bottom) Venn diagram displaying the overlap between each treatment condition. (B) Dendrogram displaying the relationship between treatment conditions and individual time points. Relationship was determined by hierarchical clustering of significantly altered genes based on TNseq log_2_ fold change at each time point. (C) Correlation between individual time points and conditions with each Varimax loading. (D) Heatmap of genes significantly associated with a Varimax dimension. Signal is based on TNseq log_2_ fold change. Boxes indicate genes significantly associated with dimensions. HRZE data were obtained from reference 36.

Having validated the accuracy of the TNseq data, we analyzed the composite data set to understand more broadly how bacterial functions alter drug efficacy. Again, we included a previously generated HRZE treatment condition, which was produced using identical methodology (36). Compared to the pretreatment time point, the number of mutants identified with altered abundance varied for each antibiotic condition (Fig. 5A and Table S4). The majority of mutations only had significantly altered susceptibility to a single agent, while a smaller subset had effects under multiple conditions (Fig. 5A). The largest overlap, 13 genes, was observed between INH and EMB, two drugs that inhibit cell wall synthesis by interrupting mycolate or arabinogalactan production. Similarities between conditions were also evident upon hierarchical clustering of significantly altered genes (Fig. 5B). Conditions clustered primarily based on regimen. Higher-order similarities based on mechanism of action were also seen, as the cell wall inhibitors (INH and EMB) were found in a branch of the dendrogram distinct from the other treatments. In addition, PZA clustered closely with HRZE, suggesting that the bactericidal activity of the combination regimen is largely driven by PZA.

While these simple comparisons indicated that each treatment generally selected a distinct set of mutants, we sought to clearly define bacterial functions that were selectively affected by each treatment. We therefore devised a multidimensional analysis to identify the bacterial genes that are most responsible for defining the treatments. Principal-component analysis (PCA) was applied to transposon insertion counts of genes across conditions to map them onto orthogonal axes (linear combinations of conditions). We then performed a Varimax rotation (37) to maximally realign the top principal components with treatment conditions, resulting in six abstract dimensions that differentiate the antibiotics based on their effects on conditional gene essentiality. All treatment groups were assigned to a distinct dimension, except for PZA and HRZE, which were similar enough to share one (Fig. 5C). This analysis also identified a clear inverse correlation between INH treatment and the untreated condition that we previously inferred (Fig. 3C). The bacterial genes most closely aligned with each Varimax dimension were identified based on their rotated PCA loadings, and the significance of these associations was determined using an approach called projection resampling (see Materials and Methods). This analysis identified between 2 and 14 genes that are significantly associated with individual treatment conditions (Fig. 5D and Table S5). For example, mutations in an operon consisting of *ppe1*, *rv0097*, *fcoT*, *fadD10*, and *nrp* were found to increase survival in the presence of INH, an effect that is consistent with previous work (35). Increased abundance of mycobactin mutants distinguishes EMB from the other treatment conditions. Genes associated with the RIF dimension include previously validated genes *rv1184c*, *rv3822*, and *cmaA2* (Fig. 4). Seven of these eight genes are involved in cell wall, lipid, or arabinan metabolism (*pks2*, *phoR*, *mmaA3*, *cmaA2*, *ephD*, *rv1184c*, and *rv3822*) (38–43), suggesting that the permeability of the mycobacterial envelope is the primary determinant of RIF activity during infection. The PZA/HRZE dimension is associated with *pncA*, the activator of the prodrug PZA, as well as mutations in the *ppe51* gene that is involved in glycerol/glucose uptake (44) and was previously found to enhance the activity of HRZE (36) (Fig. 4). In addition, mutations in several genes dedicated to the synthesis of the cell envelope lipid, phthiocerol dimycocerosate (PDIM), decreased HRZE efficacy. The untreated dimension is associated with two genes, *grcC2* and *rv1543*, that enhance susceptibility under all drug-treated conditions and encode a polyprenyl-diphosphate synthase and a predicted fatty acyl coenzyme A (acyl-CoA) reductase, respectively.

10.1128/mSystems.00396-20.9 TABLE S5 Genes significantly associated with Varimax dimensions. List of genes significantly associated with individual Varimax dimensions (Q-value <0.025). “Mean insertion counts,” average insertions counts at all TA dinucleotide sites in the open reading frame (ORF) for individual dimensions. “LFCs,” log fold changes comparing normalized insertion counts at all TA sites in the ORF for an individual dimension to the mean insertion counts of all six dimensions. “*P* adjusted,” significance adjusting for multiple tests. “V,” Varimax dimension. Treatment associations: V1, INH all time points; V2, untreated all time points; V3, PZA/HRZE all time points; V4, RIF all time points; V5, EMB 1 and 2.5 weeks; V6, EMB 5 weeks. Green = positive LFC. Red = negative LFC. Bold = Qval <0.025. Download Table S5, XLSX file, 0.02 MB. Copyright © 2020 Bellerose et al. 2020 Bellerose et al. This content is distributed under the terms of the Creative Commons Attribution 4.0 International license.

### Many susceptibility phenotypes are specific to the *in vivo* environment.

To evaluate the importance of the infection environment in shaping the mechanisms of drug susceptibility, we investigated whether mutations found to alter efficacy in animals also had an effect under standard culture conditions. We first compared the mutants found in our *in vivo* study with those previously found to alter the MICs of INH, EMB, or RIF *in vitro* using an analogous TNseq approach (25). We observed a small but significant overlap of genes associated with INH (overlap of 8 genes between 68 *in vivo* and 90 *in vitro*, *P = *0.0004), EMB (overlap of 4 genes between 54 *in vivo* and 67 *in vitro*, *P = *0.02), and RIF (overlap of 10 genes between 59 *in vivo* and 75 *in vitro*, *P = *4 × 10^−7^) treatment, identifying functions that alter treatment efficacy both *in vitro* and during infection. Despite these similarities, the majority of mutations were predicted to alter either *in vivo* efficacy or *in vitro* MIC values, but not both.

**FIG 6:**
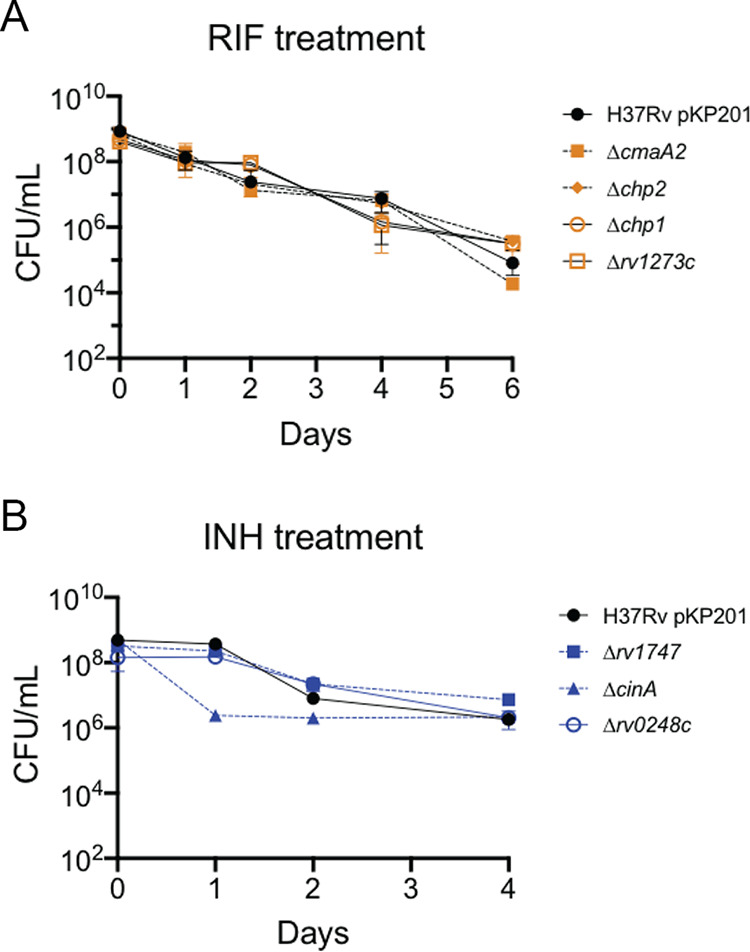
Rate of killing of mutants *in vitro*. CFU of H37Rv and deletion mutants after RIF treatment at 0.5 μg/ml (A) or INH treatment at 0.6 μg/ml (B). Mean and standard deviation from triplicates are plotted.

To more directly quantify *in vitro* effects, we took advantage of our deletion mutant set (Fig. 4). Each mutant was exposed to the antibiotic that resulted in the most differential selection for that strain *in vivo* (RIF or INH), and both the MIC_50_ and rate of killing were determined *in vitro*. While MIC differences between wild type and three mutants met statistical significance, none differed by more than 2-fold. (Table 1). When the rate of killing was measured, no differences were observed under RIF treatment. In INH treatment only a single mutant, the Δ*cinA* strain, displayed increased killing that was consistent with the *in vivo* phenotype (Fig. 6). Thus, consistent with the TNseq comparison, this analysis indicated that many of the mutations that alter *in vivo* drug efficacy have little effect during *in vitro* culture.

**TABLE 1:** Antibiotic susceptibility of deletion strains *in vitro*

Strain	IC_50_ (μg/ml ± SD)^*a*^	
	INH	RIF
H37Rv	0.03 ± 0.002	0.0033 ± 0.0003
Δ*rv0248c*	0.04 ± 0.005*	0.0035 ± 0.0002
Δ*cmaA2*	0.03 ± 0.001	0.0025 ± 0.0003*
Δ*rv1174c*	0.03 ± 0.001	0.0033 ± 0.0004
Δ*rv1184c*	0.03 ± 0.001	0.0029 ± 0.0007
Δ*rv1273c*	0.03 ± 0.005	0.0032 ± 0.0006
Δ*rv1747*	0.03 ± 0.004	0.0028 ± 0.0009
Δ*ppe51*	0.05 ± 0.005*	0.0041 ± 0.0001*
Δ*rv3822*	0.03 ± 0.002	0.0030 ± 0.0003

a IC_50_ = mean of three individual experiments. *, *P *< 0.05.

### Natural variants in efficacy-altering genes are associated with drug resistance.

**FIG 7:**
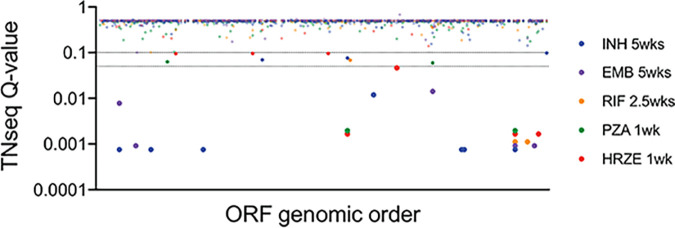
Comparison between *in vivo* susceptibility and association with clinical resistance. Three hundred twenty-eight genes associated with clinical resistance are plotted by genomic order (*x* axis), and Q-values from TNseq conditions are indicated (*y* axis). Dashed lines indicate Q-values <0.05 and <0.01. TNseq hits overlapping genes associated with clinical resistance are indicated by filled circles.

In the mouse model, we identified many genes that have the capacity to alter antibiotic efficacy (Fig. 3B and Table S4). Reasoning that naturally occurring polymorphisms in these genes might be selected in the context of antibiotic treatment, we investigated if there was overlap between genes identified in our mouse studies and those previously found to contain resistance-associated single nucleotide polymorphisms (SNPs) in clinical isolates. We utilized data from three published GWAS (19, 20, 22) that identified genes that are subject to convergent evolution in drug-resistant isolates. We compared these genes to the loss-of-function mutations that we found to either increase or decrease antibiotic killing in the mouse, since naturally occurring polymorphisms could increase, decrease, or alter the functions of these genes. Of the 328 genes identified by GWAS, 14 were also identified in our TNseq study with a Q-value of < 0.05, and 21 overlapped with a Q-value less than 0.1 (Fig. 7 and Table 2). Genes known to alter drug sensitivity (*pncA*) and tolerance (*glpK*) were identified, along with a number of genes that have not been shown to influence drug efficacy. For example, we find that disruption of the nonribosomal peptide synthase gene, *nrp*, produces tolerance to INH in the mouse, which likely explains the association of *nrp* variants with clinical INH resistance (19). Similarly, loss of *pks2* (polyketide synthase) function reduced RIF activity in mice, and SNPs in the *pks2* gene are associated with clinical ofloxacin resistance (20). As individual resistance traits in multidrug-resistant isolates are linked, these observations are consistent with *pks2* mutations contributing to this phenotype either by increasing RIF tolerance or by influencing the effects of multiple drugs, including fluoroquinolones, which were not tested in the mouse. While the overlap between these data sets was relatively small, this analysis allowed us to causally implicate variants in at least 14 M. tuberculosis genes in the evolution of drug resistance. As many of the efficacy-altering mutations found in the mouse model have little effect *in vitro* (Table 1 and Fig. 6), we speculate that the effects of these natural variants may not be apparent under similar *in vitro* conditions. If so, these variants could represent cryptic determinants of treatment outcome that preferentially alter drug efficacy during infection.

**TABLE 2:** Genes with SNPs associated with resistance and altered susceptibility phenotypes *in vivo*^*a*^

Gene	Phenotype(s)	GWAS data set	GWAS phenotype
*rv0101* (*nrp*)	**INH**; *EMB*	Hicks et al. (19)	INH
*rv0244c* (*fadE5*)	**EMB**	Zhang et al. (20)	KAN
*rv0353* (*hspR*)	*INH*	Farhat et al. (22)	RIF; INH; EMB; CAP
*rv0859* (*fadA*)	*INH*	Zhang et al. (20)	OFX; KAN
*rv2043c* (*pncA*)	**PZA**; **HRZE**	Farhat et al. (22)	AMI; CAP; EMB; ETA; INH; KAN; MXF; PZA; RFB; RIF; STR; LIN
*rv2344c* (*dgt*)	*INH*	Hicks et al. (19)	INH
*rv2571c*	**HRZE**	Farhat et al. (22)	KAN; CAP; AMI; ETA; PZA; STR; RFB
*rv2942* (*mmpL7*)	*INH*; *EMB*	Farhat et al. (22)	RIF
*rv3211* (*rhlE*)	*INH*	Hicks et al. (19)	INH
*rv3267*	*INH*	Hicks et al. (19)	INH
*rv3696c* (*glpK*)	**INH**; **EMB**; **RIF**; **PZA**; **HRZE**	Farhat et al. (22)	AMI; INH; KAN; RFB; RIF; CAP; LIN; EMB; ETA; PZA
*rv3825c* (*pks2*)	**RIF**	Zhang et al. (20)	OFX
*rv3859c* (*gltB*)	EMB	Farhat et al. (22)	STR
*rv3877* (*eccD1*)	*HRZE*	Zhang et al. (20)	CAP
rv0560c*	*PZA*	Hicks et al. (19)	INH
rv0600c*	*HRZE*	Zhang et al. (20)	KAN
*rv1282c* (*oppC*)*	**HRZE**	Hicks et al. (19)	INH
*rv1330c* (*pncB1*)*	*INH*	Farhat et al. (22)	KAN; CAP; ETA; RIF; STR
*rv1860* (*apa*)*	*HRZE*	Farhat et al. (22)	STR, RIF
*rv2080* (*lppJ*)*	*RIF*	Zhang et al. (20)	ETH; KAN
*rv3919c* (*gid*)*	*INH*	Farhat et al. (22)	EMB; INH; MXF; PZA; RFB; RIF; STR; CAP; LIN; ETA; KAN

a Symbols and abbreviations: bold, overrepresented in drug-treated TNseq samples; italic, underrepresented in drug-treated TNseq samples; GWAS phenotype, the drug resistance pattern associated with SNPs; INH, isoniazid; EMB, ethambutol; RIF, rifampin; PZA, pyrazinamide; KAN, kanamycin; CAP, capreomycin; OFX, ofloxacin; MXF, moxifloxacin; AMI, amikacin; ETA, ethionamide; STR, streptomycin; RFB, rifabutin; LIN, linezolid; *, Q-value <0.1.

## DISCUSSION

Many studies investigating antibiotic efficacy and new drug target discovery are performed *in vitro*. While it is possible to change discrete aspects of the culture conditions to mimic individual stresses (25, 45–48), these models do not fully recapitulate the complex environment encountered by the bacterial population during infection. In this study, we identified genes important for bacterial survival under antibiotic pressure in the mouse model of TB, where the bacteria grow intracellularly (49) in the presence of a fully functional adaptive immune response. By collecting data across several time points, we were able to discern a number of new insights into the processes necessary to sustain an infection and persist through antibiotic treatment.

This time-resolved study provides the most detailed assessment of M. tuberculosis genes necessary to persist in the mouse model to date, identifying 562 genes (see Table S2 in the supplemental material). Our data are consistent with previous studies and identified a large number of known virulence factors. We also identified 231 genes that were not found in previous TNseq studies, reflecting the increased accuracy of UMI-based quantification of transposon insertions and the increased number of replicates and time points. These included functions already known to be important, such as a number of genes encoded by a large genomic region dedicated to cholesterol catabolic functions (*kshA*, *rv3538*, *rv3549c*, *echA20*, *rv3557c*, *rv3562*, *rv3570c*, and *rv3575c*) (50, 51). Similarly, several additional genes related to type VII protein secretion were identified: *cyp143*, *ppe27*, and *esxN* are components of the ESX5 system (52), *esxW* is homologous to ESX substrates and has been associated with TB transmission (53), and *rv3866* (*espG*) is a component of the ESX1 system (54). A number of novel functions were identified as well. For example, we found genes encoding a succinate dehydrogenase complex (*sdhA*, *sdhB*, and *sdhD*), the proton-translocating NADH dehydrogenase (*nuoE* and *nuoK*), and the Mce3 transporter that is homologous to lipid importers (*mce3A*, *mce3B*, *mce3C*, and *lprM*). Overall, this data set enhances our understanding of the genomic requirements for infection.

When infected animals were treated with antibiotics, we found only a small number of genes that broadly alter drug efficacy. These included *glpK*, which is necessary for glycerol metabolism and has been shown to alter the effect of HRZE *in vivo* (36) and several drugs *in vitro* (55). Similarly, mutation of a putative fatty-acyl-CoA reductase encoded by *rv1543* broadly sensitizes the bacterium to different drugs. These observations highlight the importance of primary metabolic functions in general alterations in drug sensitivity. A much larger collection of mutations produced relatively drug-specific effects (Fig. 5). We used a PCA-based strategy to find condition-defining mutations, similar to an approach previously applied to TNseq data (56). By designing a new statistical framework to assess the significance of gene-condition associations, we identified sets of mutants that can be used to infer the primary mechanisms that determine the efficacy of some antibiotics. For example, nearly all of the genes associated with RIF treatment are likely to be involved in cell wall formation, such as acyltransferases Rv1184c and Rv3822 and cyclopropane synthase CmaA2. While *rv1184c* and *rv3822* mutants are more susceptible to RIF, mutations in *cmaA2* result in increased survival, indicating that changes in permeability can affect RIF efficacy in multiple ways. More generally, the abundance of cell wall-modifying enzymes indicates that permeability is an important determinant of RIF efficacy during infection, which is consistent with previous *in vitro* observations (57–59). Similarly, mutants in the mycobactin biosynthesis pathway were overrepresented specifically post-EMB treatment, indicating a role for iron utilization in EMB efficacy. Finally, the specific correlation between *in vivo* fitness and drug efficacy for INH (Fig. 3C and Fig. 5C), a drug known to be affected by growth rate *in vitro* (10, 35), suggested that INH is preferentially affected by the decreased replication rate of the bacterium during infection.

Drug efflux may also produce drug-selective effects. For example, Rv1273c is predicted to be a multidrug transporter based on sequence homology (60), and we found this mutant was hypersusceptible only to RIF. Similarly, loss of the ABC transporter encoded by *rv1747* specifically increased INH susceptibility (Fig. 4). Despite these *in vivo* effects, we found no evidence that mutating these genes altered drug susceptibility *in vitro*, suggesting that both systems are regulated. Indeed, Rv1747 is an ABC transporter that is controlled via phosphorylation by PknF (61), indicating a potential mechanism of inducing INH tolerance in response to environmental cues. In contrast, Rv1273c expression is increased in clinical isolates (62), leading to the hypothesis that this may be an inducible efflux pump, similar to a previously identified mycobacterial drug efflux system that is expressed during intracellular growth (15).

While we did not globally assess the effect of transposon mutations on antibiotic efficacy *in vitro*, we compared our *in vivo* data set to a previous TNseq study (25) and directly measured *in vitro* effects for a selection of mutants. Both efforts indicated that many of the efficacy-altering mutations that we identified in the mouse model have a minimal effect on *in vitro* MIC or rate of killing. This observation has important implications, as it suggests the possibility that many genetic variants that alter drug activity do not produce an effect that is measurable in standard drug susceptibility testing (DST). Recent evidence showing that very small MIC alterations predict human treatment outcome (17) highlights the potential importance of these variants.

Genetic variants that are selected by drug exposure can be identified via GWAS approaches using the thousands of available whole-genome sequences from M. tuberculosis clinical isolates (19, 20, 22). While these data are immediately useful for genotypic drug susceptibility assessment (63, 64), the functional roles played by the majority of these variants remain unknown. In this work, we leveraged our TNseq data to identify a number of variants that are likely to directly alter drug efficacy, suggesting new mechanisms that are relevant to treatment outcome. However, the relatively modest overlap between the TNseq and GWAS data sets was also notable. It is possible that this observation indicates that only a small fraction of the variants identified by GWAS directly alter drug efficacy. However, this conclusion should be approached with caution, as there are significant physiological differences between human and mouse TB. Furthermore, the TNseq approach only assesses the effect of loss-of-function mutations in the context of a splenic infection, raising the possibility that some lung-specific effects could have been missed. Thus, the ultimate functional assessment of natural genetic polymorphisms still requires the individual investigation of each variant.

Understanding how Mycobacterium tuberculosis survives prolonged antibiotic pressure also suggests new strategies to improve treatment. Our data indicate that a large number of potential synergies exist that could be exploited to accelerate bacterial clearance. While we do not assess sterilization or ultimate “cure” in this model, rapidly eliminating viable bacteria remains an important goal. While the relatively drug-selective effects of these synergies represent a potential challenge, our data indicate that more effective regimens are possible and their development could be facilitated by this type of unbiased chemical-genetic study.

## MATERIALS AND METHODS

### Transposon sequencing.

BALB/cJ (stock no. 000651) mice were purchased from Jackson Laboratory (Bar Harbor, ME, USA). Housing and experimentation were in accordance with the guidelines set forth by the Department of Animal Medicine of University of Massachusetts Medical School and Institutional Animal Care and Use Committee and adhered to the laws of the United States and regulations of the Department of Agriculture. Eight- to 12-week-old female animals were infected with 10^6^ CFU of a *himar1* transposon library (65) via the intravenous route. At 14 days postinfection, antibiotics were administered via drinking water at the following concentrations: 0.1 g/liter isoniazid (Sigma), 0.6 g/liter ethambutol (Sigma), 0.1 g/liter rifampin (Sigma), 15 g/liter pyrazinamide (Sigma). At indicated time points, mice were sacrificed, spleens and lungs were isolated and homogenized, and CFU was determined by plating dilutions on 7H10 agar with 10 μg/ml kanamycin. For library recovery, approximately one million CFU per mouse were plated on 7H10 agar with kanamycin (10 μg/ml). Genomic DNA was extracted and the relative abundance of each mutant was estimated and normalized as described previously (30). Statistical analysis of log_2_ fold change (log_2_FC) in normalized counts between conditions was performed by resampling (66). Hierarchical clustering [using *hclust()* in R, with average-linkage clustering] was applied to vectors of log_2_FC for each gene across all conditions. PCA and Varimax rotation were performed on log fold changes (LFCs) using the procedures *prcomp()* and *varimax()* in R, where the LFC for each condition was calculated as the log_2_ of the ratio of the mean insertion count in that condition relative to the grand mean across all conditions.

### M. tuberculosis strains and culturing.

M. tuberculosis H37Rv was grown in Middlebrook 7H9 medium containing oleic acid-albumin-dextrose-catalase (OADC), 0.2% glycerol, and 0.05% Tween 80 and grown with shaking (200 rpm) at 37°C. Hygromycin (50 μg/ml) or kanamycin (20 μg/ml) was added when necessary. All work with M. tuberculosis adhered to the CDC-NIH guide for biosafety in microbiological and biomedical laboratories (67). Deletion strains were constructed by allelic exchange as previously described (68), and this work adhered to NIH guidelines for research involving recombinant DNA molecules. Genes were replaced by the vector pKM464 carrying one of seven unique q-Tag sequences to identify each mutant for deep sequencing (69) (see Table S6 in the supplemental material for strain details).

10.1128/mSystems.00396-20.10 TABLE S6 Strain and primer list. List of strains constructed in these studies and primer sequences used for Illumina sequencing of mutants in pooled infections. Purple, annealing sequence. Red, unique molecular counter. Blue, Illumina index. Download Table S6, XLSX file, 0.01 MB. Copyright © 2020 Bellerose et al. 2020 Bellerose et al. This content is distributed under the terms of the Creative Commons Attribution 4.0 International license.

### *In vivo* antibiotic susceptibility.

Mice were infected with pools of strains at equal ratios via the intravenous route (10^6^ total CFU/mouse) or aerosol route (500 to 1,000 CFU/mouse). Groups of mice were treated with antibiotics, as described for the TNseq study. Treatment was administered starting at 14 days postinfection for i.v. infections and 21 days postinfection for aerosol infections. At indicated time points, approximately 10,000 CFU from the spleen homogenate of each mouse was plated on 7H10 agar. Genomic DNA was extracted for sequencing as described previously (30). Sequencing libraries spanning the variable region of each q-Tag were generated using PCR primers binding to regions common among all q-Tags, similarly to previously described protocols (70) (see Table S6 for primer details). During this PCR, a unique molecular counter was incorporated into the sequence to allow for the accurate counting of input templates and account for PCR jackpotting. The libraries were sequenced to 1,000-fold coverage on an Illumina NextSeq platform using a 150-cycle Mid-Output kit with single-end reads. Total abundance of each mutant in the library was determined by counting the number of reads for each q-Tag with a unique molecular counter. Relative abundance of each mutant in the pool was then calculated by dividing the total abundance of a mutant by the total abundance of reads for wild-type H37Rv. This value was then normalized to the relative abundance at the pretreatment time point to obtain the final relative abundance for each mutant in the pool. Statistical significance was determined by unpaired *t* test with Benjamini-Hochberg multiple testing correction.

Competition infections were performed by infecting mice with a 1:1 mixture of Δ*rv1273c* and H37Rv strains (chromosomally integrated plasmid pJEB402 encoding kanamycin resistance) or a 1:1 mixture of Δ*rv1273c* complement strain and kanamycin-resistant H37Rv via the aerosol route (500 to 1,000 CFU/mouse). After 21 days postinfection, RIF was administered to groups of mice. At indicated time points, mice were sacrificed and CFU in lung and spleen homogenate was determined by plating on 7H10 agar containing either hygromycin (50 μg/ml) or kanamycin (20 μg/ml).

### *In vitro* antibiotic susceptibility.

For MIC testing, bacteria were inoculated to a starting optical density at 600 nm (OD_600_) of 0.05 in 96-well plates with 7H9 medium containing OADC, 0.2% glycerol, and 0.05% Tween 80. Isoniazid and rifampin were used at 0.4 and 0.05 μg/ml, respectively, and serially diluted 2-fold for a total of 6 dilutions. Growth was monitored by OD_600_, and conditions were assessed in triplicate. Fifty percent inhibitory concentration (IC_50_) was determined by plotting OD versus concentration of antibiotic and plotting a curve using [inhibitor] versus response model.

For kill curves, bacteria were inoculated to a starting OD_600_ of 0.05 in inkwells containing 7H9 medium containing OADC, 0.2% glycerol, and 0.05% Tween 80. At an OD_600_ of ∼0.8 to 1.0, antibiotics were added to a final concentration of 0.6 and 0.5 μg/ml for isoniazid and rifampin, respectively. At indicated time points, samples from the cultures were taken and CFU/ml was determined by plating on 7H10 agar with 50 μg/ml hygromycin. Conditions were assessed in triplicate.

### Projection resampling.

In order to identify genes significantly associated with individual Varimax dimensions, we devised a sampling-based version of the permutation test. We redistributed the original observations (insertion counts at TA sites) representing individual experimental conditions over the Varimax dimensions, weighted by the loadings of each condition on each Varimax dimension. Since loading coefficients (from PCA plus Varimax rotation) could be positive or negative, we used the squares of the loading coefficients as weights, normalized by the sum of squares across all conditions (so they sum to 1). Let *W_c_*_,_*_v_* be the matrix of weights (normalized squares of loadings) of each condition *c* (i.e., drug) projected onto each Varimax dimension *v*. If the loading coefficients are λ*_c_*_,_*_v_*, then *W_c_*_,_*_v_* = λ^2^*_c_*_,_*_v_*/Σ*_j_* λ^2^*_j_*_,_*_v_*.

For a given gene *G*, we collected the normalized insertion counts at all TA sites in the gene across all conditions (drug treatments), averaging over replicates. We call this matrix of observations *O_i_*_,_*_c_*, where *i* indexes the TA sites in the gene. We computed projected counts, *P_i_*_,_*_v_*, for each TA site *i* on each Varimax dimension *v* as a weighted combination. In matrix notation, *P*_(_*_i_*_,_*_v_*_)_ = *O*_(_*_i_*_,_*_c_*_)_ × *W*_(_*_c_*_,_*_v_*_)_.

We then used resampling (66) (as a nonparametric permutation test) to determine the degree to which the projected counts were unusually high (or low) in each Varimax dimension, compared to all the others. Let *D* be the Varimax dimension of interest for testing the association of gene *G*. The redistributed observations for *G* were divided into two groups, A (those counts associated with dimension *D*) and B (those counts not associated with dimension *D*). Finally, the significance of the difference in mean counts in A versus B was determined by a permutation test, where a null distribution on the difference in means was generated by randomly permuting the counts between groups A and B 10,000 times, from which a *P* value for the association of gene *G* with dimension *D* was derived. *P* values were adjusted *post hoc* by the Benjamini-Hochberg procedure (71) for multiple test correction (to limit the false-discovery rate to 5%).

### Data availability.

Raw sequencing data in FASTQ and processed formats are available for download from NCBI Gene Expression Omnibus (GEO) under accession number GSE154627. Data processing pipelines used in this work are available on GitHub: https://github.com/sassettilab/Bellerose_et_al_mSystems_TnSeq_analyses.
