# Supplementary figures and images for: Distinct Bacterial Pathways Influence the Efficacy of Antibiotics against Mycobacterium tuberculosis
Source: mSystems. 2020 Aug 4;5(4):e00396-20. doi: 10.1128/mSystems.00396-20 (PMC7406225; doi:10.1128/mSystems.00396-20)

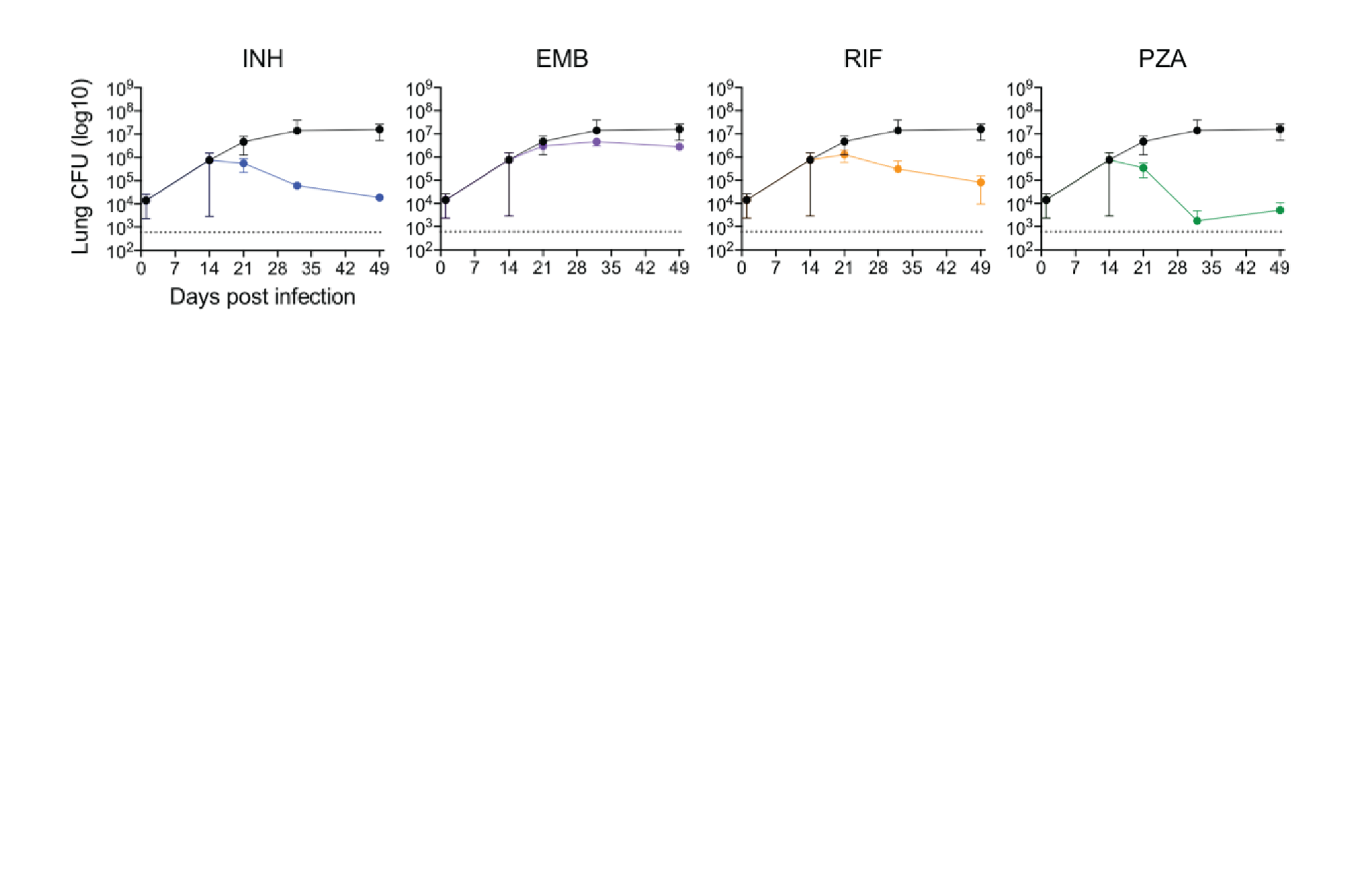

Supplement: FIG S1 [file mSystems.00396-20-sf001.tif]

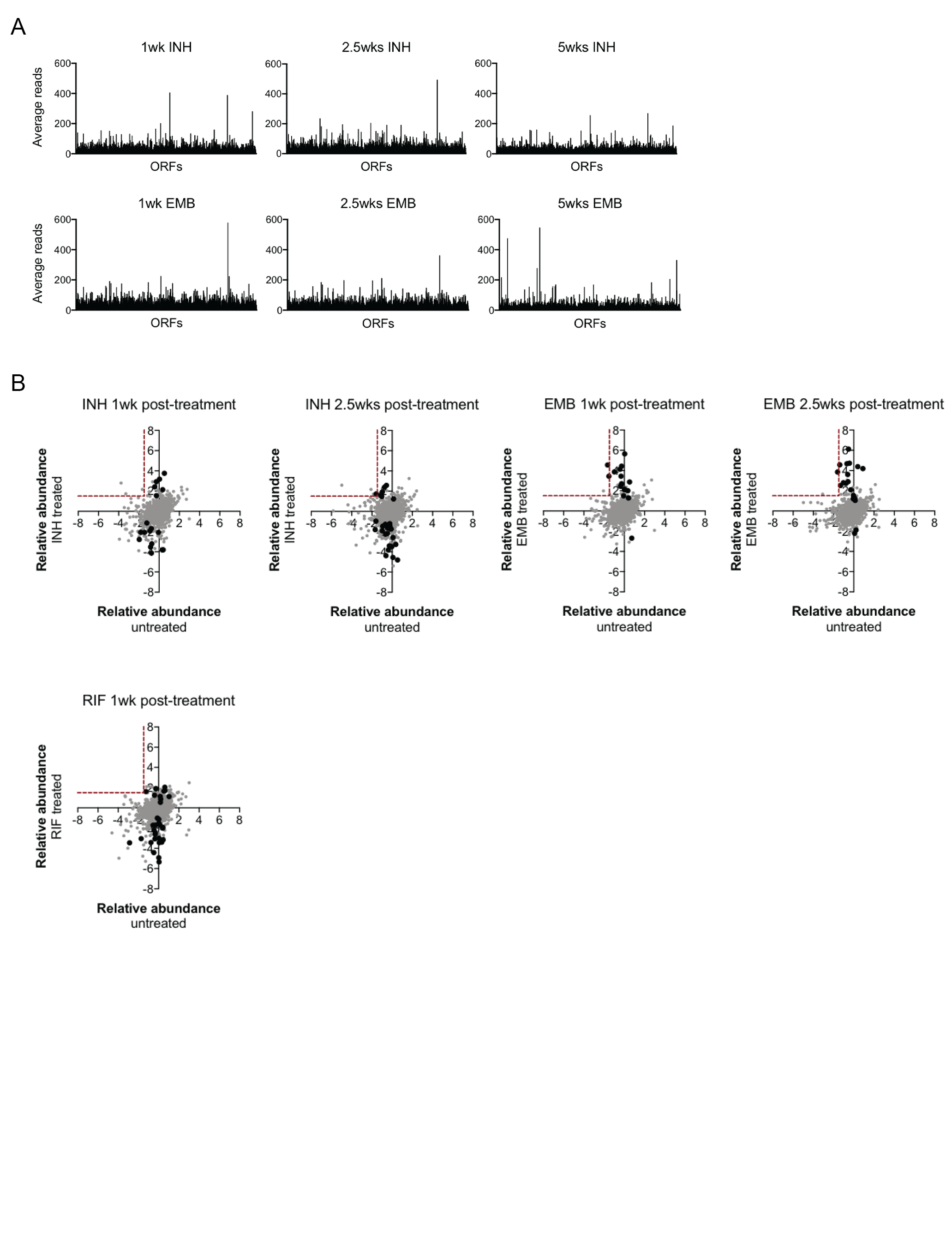

Supplement: FIG S2 [file mSystems.00396-20-sf002.tif]

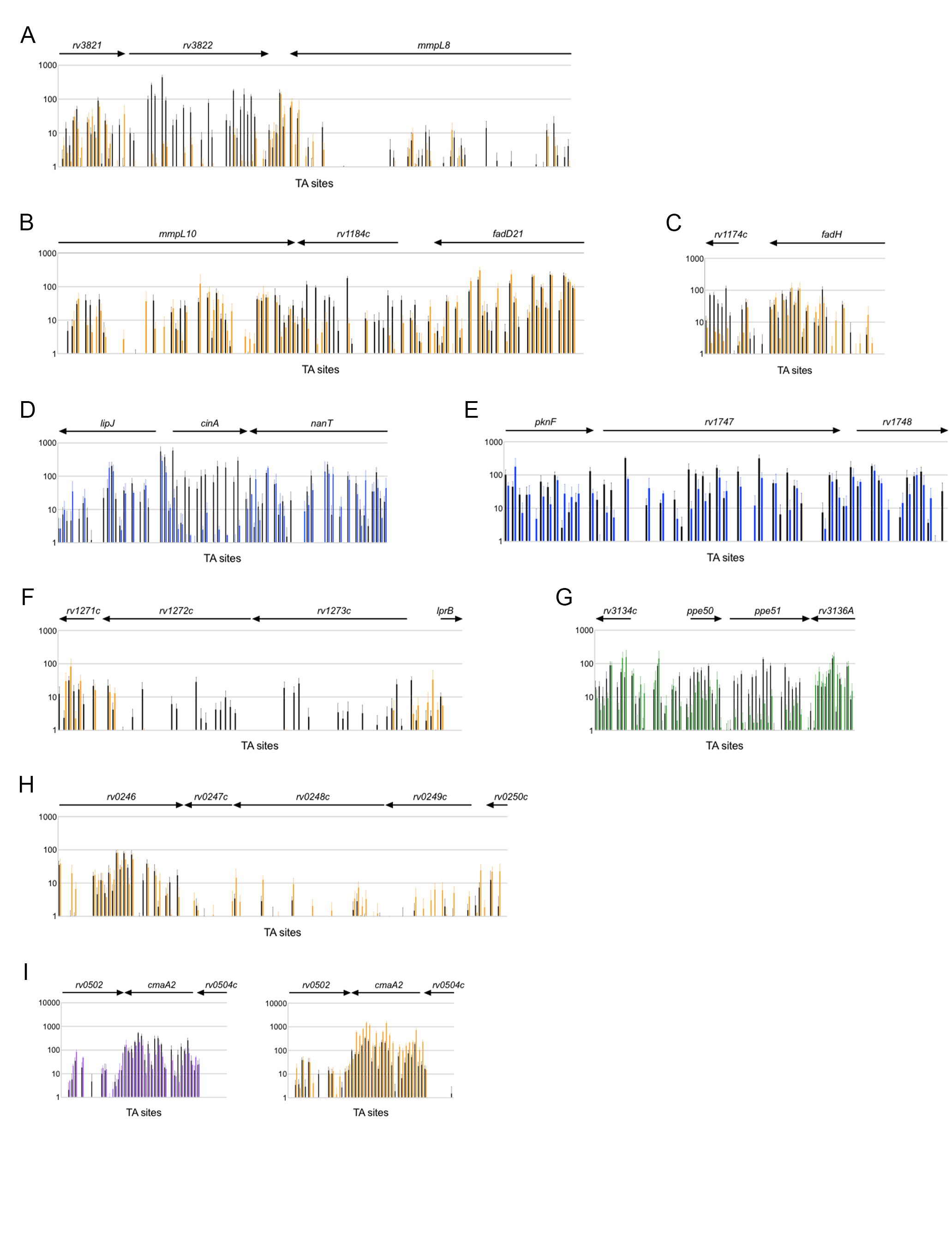

Supplement: FIG S3 [file mSystems.00396-20-sf003.tif]

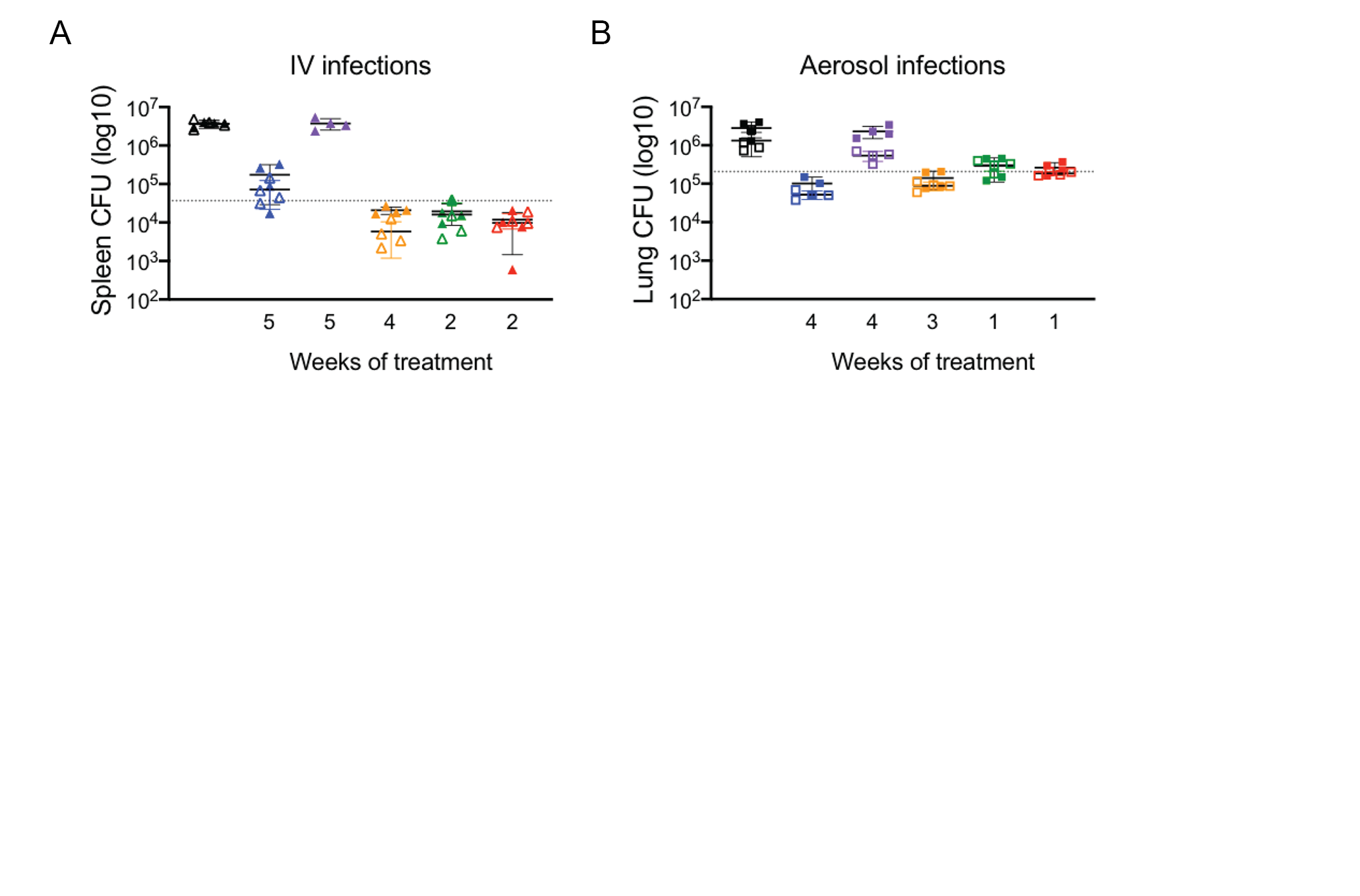

Supplement: FIG S4 [file mSystems.00396-20-sf004.tif]
